# Supplementary material for: Chimney vs. Fenestrated Endovascular vs. Open Repair for Juxta/Pararenal Abdominal Aortic Aneurysms: Systematic Review and Network Meta-Analysis of the Medium-Term Results
Source: J Clin Med. 2022 Nov 16;11(22):6779. doi: 10.3390/jcm11226779 (PMC9693018; doi:10.3390/jcm11226779)
Supplement: Supplementary file 1 [file jcm-11-06779-s001.zip › jcm-2017913-supplementary.pdf]

Appendix SA. Search strategy

Medline (via pubmed)

#	Searches	Results
1	“JAAA” [tiab] OR “jrAAA” [tiab] OR “juxtarenal abdominal aortic aneurysm*” [tiab] OR “juxtarenal aortic aneurysm*” [tiab] OR „juxtarenal aneurysm” [tiab] OR “pararenal abdominal aortic aneurysm” [tiab] OR “pararenal aortic aneurysm” [tiab] OR “pararenal aneurysm” OR “short neck* aneurysm*” [tiab] OR (“short neck” AND aneurysm*) OR “complex abdominal aortic aneurysm*” [tiab] OR “cAAA” [tiab] OR “complex aortic aneurysm” [tiab] or “complex aneurysm” [tiab]	996
2	"open surger*" [tiab] OR "open repair*" [tiab] OR "open procedure*" [tiab] OR "open technique*" OR "open surgical repair*" OR "open aneurysm repair*" [tiab] OR "open aortic aneurysm repair*" [tiab] OR "open aortic repair*" [tiab] OR "OAR" [tiab] OR “OSR” [tiab]	36,665
3	“FEVAR” [tiab] OR “f-EVAR” [tiab] OR “fenestrated endograft*” [tiab] OR “fenestrated EVAR” [tiab] OR “fenestrated endovascular aneurysm repair*” [tiab] OR "fenestrated stent graft*" [tiab] OR “fenestrated endovascular aortic repair*” [tiab] OR “fenestrated endovascular repair*” [tiab]	854
4	”BEVAR” [tiab] OR ”b-EVAR” [tiab] OR ”branched endograft*”	745

	[tiab] OR ”branched EVAR” [tiab] OR "branched endovascular aneurysm repair*" [tiab] OR "branched stent graft*" [tiab] OR ”branched endovascular aortic repair*”[tiab] OR “branched endovascular repair*“ [tiab]	
5	”F-BEVAR” [tiab] OR ”fenestrated branched endograft*” [tiab] OR ”fenestrated branched EVAR” [tiab] OR ”fenestrated branched endovascular repair*” [tiab] OR ”fenestrated branched endovascular aneurysm repair*” [tiab] OR ”fenestrated branched stent graft*” [tiab] OR ”fenestrated branched endovascular aortic repair*” [tiab]	168
6	#3 OR #4 OR #5	1,438
7	“ChEVAR” [tiab] OR “ch-EVAR” [tiab] OR “chimney endograft*” [tiab] OR “chimney EVAR” [tiab] OR “parallel graft*” [tiab] OR “chimney endovascular repair*” [tiab] OR “chimney technique*” [tiab] OR “chimney endovascular aneurysm repair” [tiab] OR “chimney stent graft*” [tiab] OR “parallel graft*” [tiab] OR snorkel OR periscope	1,415
8	“EVAR” [tiab] OR “endovascular repair” [tiab] OR “endovascular aneurysm repair*” [tiab] OR “endovascular aortic repair*” [tiab]	13,669
9	"physician modified" [tiab] OR "PMFG" [tiab] OR "Physician-modified fenestrated endovascular" [tiab] OR "PMEG" [tiab] OR "physician modified endovascular graft" [tiab] OR "physician-modified endograft*" [tiab]	257
10	#2 AND (#6 OR #7 OR #8 OR #9)	4,030

11	#6 AND (#7 OR #8 OR #9)	1,128
12	#7 AND (#8 OR #9)	423
13	#8 AND #9	109
14	#1 AND (#10 OR #11 OR #12 OR #13)	390

Scopus

#	Searches	Results
1	JAAA” OR “jrAAA” OR “juxtarenal abdominal aortic aneurysm*” OR “juxtarenal aortic aneurysm*” OR “juxtarenal aneurysm” OR “pararenal abdominal aortic aneurysm” OR “pararenal aortic aneurysm” OR “pararenal aneurysm” OR “short neck* aneurysm*” OR (“short neck” AND "aneurysm*") OR “complex abdominal aortic aneurysm*” OR “cAAA” OR “complex aortic aneurysm” OR “complex aneurysm”	2,805
2	"open surger*" OR "open repair*" OR "open procedure*" OR "open technique*" OR "open surgical repair*" OR "open aneurysm repair*" OR "open aortic aneurysm repair*" OR "open aortic repair*" OR "OAR" OR “OSR”	56,669
3	”FEVAR” OR “f-EVAR” OR “fenestrated endograft*” OR “fenestrated EVAR” OR “fenestrated endovascular aneurysm repair*” OR "fenestrated stent graft*" OR “fenestrated endovascular aortic repair*” OR “fenestrated endovascular repair*”	934
4	”BEVAR” OR ”b-EVAR” OR ”branched endograft*” OR ”branched EVAR” OR "branched endovascular aneurysm repair*" OR "branched stent graft*" OR ”branched endovascular aortic repair*” OR “branched endovascular repair*“	821
5	”F-BEVAR” OR ”fenestrated branched endograft*” OR ”fenestrated branched EVAR” OR ”fenestrated branched	183

	endovascular repair*” OR ”fenestrated branched endovascular aneurysm repair*” OR ”fenestrated branched stent graft*” OR ”fenestrated branched endovascular aortic repair*”	
6	#3 OR #4 OR #5	1,558
7	“ChEVAR” OR “ch-EVAR” OR “chimney endograft*” OR “chimney EVAR” OR “parallel graft*” OR “chimney endovascular repair*” OR “chimney technique*” OR “chimney endovascular aneurysm repair” OR “chimney stent graft*” OR “parallel graft*” OR snorkel OR periscope	2,656
8	”EVAR” OR “endovascular repair” OR “endovascular aneurysm repair*” OR “endovascular aortic repair*”	20,018
9	"physician modified" OR "PMFG" OR "Physician-modified fenestrated endovascular" OR "PMEG" OR "physician modified endovascular graft" OR "physician-modified endograft*"	351
10	#2 AND (#6 OR #7 OR #8 OR #9)	5,220
11	#6 AND (#7 OR #8 OR #9)	1,282
12	#7 AND (#8 OR #9)	513
13	#8 AND #9	119
14	#1 AND (#10 OR #11 OR #12 OR #13)	702

Web of science

#	Searches	Results
1	TS=(“JAAA” OR “jrAAA” OR “juxtarenal abdominal aortic aneurysm*” OR “juxtarenal aortic aneurysm*” OR “juxtarenal aneurysm” OR “pararenal abdominal aortic aneurysm” OR “pararenal aortic aneurysm” OR “pararenal aneurysm” OR “short neck* aneurysm*” OR (“short neck” AND "aneurysm*") OR “complex abdominal aortic aneurysm*” OR “cAAA” OR “complex aortic aneurysm” OR “complex aneurysm”)	1,056
2	TS=("open surger*" OR "open repair*" OR "open procedure*" OR "open technique*" OR "open surgical repair*" OR "open aneurysm repair*" OR "open aortic aneurysm repair*" OR "open aortic repair*" OR "OAR" OR “OSR”)	37,164
3	TS=(”FEVAR” OR “f-EVAR” OR “fenestrated endograft*” OR “fenestrated EVAR” OR “fenestrated endovascular aneurysm repair*” OR "fenestrated stent graft*" OR “fenestrated endovascular aortic repair*” OR “fenestrated endovascular repair*”)	1,009
4	TS=(”BEVAR” OR ”b-EVAR” OR ”branched endograft*” OR ”branched EVAR” OR "branched endovascular aneurysm repair*" OR "branched stent graft*" OR ”branched endovascular aortic repair*” OR “branched endovascular repair*“)	885
5	TS=(”F-BEVAR” OR ”fenestrated branched endograft*” OR	199

	”fenestrated branched EVAR” OR ”fenestrated branched endovascular repair*” OR ”fenestrated branched endovascular aneurysm repair*” OR ”fenestrated branched stent graft*” OR ”fenestrated branched endovascular aortic repair*”)	
6	#3 OR #4 OR #5	1,694
7	TS=(“ChEVAR” OR “ch-EVAR” OR “chimney endograft*” OR “chimney EVAR” OR “parallel graft*” OR “chimney endovascular repair*” OR “chimney technique*” OR “chimney endovascular aneurysm repair” OR “chimney stent graft*” OR “parallel graft*” OR snorkel OR periscope)	2,230
8	TS=(“EVAR” OR “endovascular repair” OR “endovascular aneurysm repair*” OR “endovascular aortic repair*”)	16,135
9	TS=("physician modified" OR "PMFG" OR "Physician-modified fenestrated endovascular" OR "PMEG" OR "physician modified endovascular graft" OR "physician-modified endograft*")	318
10	#2 AND (#6 OR #7 OR #8 OR #9)	4,447
11	#6 AND (#7 OR #8 OR #9)	1,383
12	#7 AND (#8 OR #9)	488
13	#8 AND #9	110
14	#1 AND (#10 OR #11 OR #12 OR #13)	451

Appendix SB. Extracted data from each study with a pre-specified proforma

- *Study characteristics:* first author; year of publication; study design (prospective or retrospective observational); type of AAA (JAAA, PAAA, or both) and intervention (ChEVAR, FEVAR, OS), study duration.
- *Study demographics:* number of participants per each treatment arm; mean age; female gender; smoking; comorbidities including hypertension, hyperlipidemia, diabetes mellitus, chronic obstructive pulmonary disease, coronary artery disease (CAD), cerebrovascular disease, chronic kidney disease (CKD); AAA diameter.
- *Periprocedural data:* for endovascular group: the number of chimney/fenestrations, stent-graft manufacturer, for open surgery group: proximal clamp position and mean proximal clamping time, operation duration, total blood loss, hospital and intensive care unit stay

Appendix SC (1). Summary of findings table showing comparison FEVAR vs open surgery for JAAA/PAAA treatment

Outcomes	Anticipated absolute effects* (95% CI)		Relative effect (95% CI)	№ of participants (studies)	Certainty of the evidence (GRADE)
	Risk with Open surgery	Risk with FEVAR			
All-cause mortality	81 per 1,000	**112 per 1,000** (83 to 140)	**OR 1.44** (1.03 to 1.86)	4006 (14 observational studies)	⊕⊕⊕◯ Moderate
Aortic-related reintervention	36 per 1,000	**240 per 1,000** (126 to 507)	**OR 8.32** (3.80 to 27.16)	1074 (11 observational studies)	⊕⊕⊕◯ Moderate
Aortic-related mortality	9 per 1,000	**6 per 1,000** (1 to 48)	**OR 0.65** (0.06 to 5.67)	920 (10 observational studies)	⊕◯◯◯ Very low
Branch/bypass occlusion/stenosis	8 per 1,000	**99 per 1,000** (22 to 468)	**OR 13.13** (2.70 to 105.20)	1194 (13 observational studies)	⊕⊕◯◯ Low
Major adverse cardiovascular event	37 per 1,000	**57 per 1,000** (19 to 183)	**OR 1.57** (0.52 to 5.88)	712 (5 observational studies)	⊕◯◯◯ Very low
New onset renal replacement therapy	14 per 1,000	**17 per 1,000** (2 to 160)	**OR 1.27** (0.13 to 13.87)	435 (6 observational studies)	⊕◯◯◯ Very low

AAA: abdominal aortic aneurysm; **EVAR:** endovascular aneurysm repair; **FEVAR:** fenestrated EVAR; **ChEVAR:** chimney EVAR; **OS:** open surgery; **JAAA/PAAA:** juxta/pararenal abdominal aortic aneurysm

Appendix SC (2). Summary of findings table showing comparison Chimney EVAR vs open surgery for JAAA/PAAA treatment

Outcomes	**Anticipated absolute effects*** (95% CI)		Relative effect (95% CI)	№ of participants (studies)	Certainty of the evidence (GRADE)
	**Risk with Open surgery**	**Risk with ChEVAR**			
All-cause mortality	81 per 1,000	**106 per 1,000** (61 to 174)	**OR 1.35** (0.74 to 2.40)	2773 (14 observational studies)	⨁⨁⨁◯ Moderate
Aortic-related reintervention	36 per 1,000	**184 per 1,000** (78 to 433)	**OR 5.95** (2.23 to 20.18)	771 (11 observational studies)	⨁⨁◯◯ Low
Aortic-related mortality	9 per 1,000	**9 per 1,000** (1 to 87)	**RR 0.99** (0.07 to 9.76)	679 (10 observational studies)	⨁◯◯◯ Very low
Branch/bypass occlusion/stenosis	8 per 1,000	**123 per 1,000** (23 to 596)	**OR 16.82** (2.79 to 176.70)	893 (13 observational studies)	⨁⨁◯◯ Low
Major adverse cardiovascular event	37 per 1,000	**210 per 1,000** (26 to 797)	**OR 6.96** (0.70 to 103.00)	449 (5 observational studies)	⨁◯◯◯ Very low
New onset renal replacement therapy	14 per 1,000	**15 per 1,000** (0 to 403)	**OR 1.09** (0.02 to 48.97)	303 (6 observational studies)	⨁◯◯◯ Very low

AAA: abdominal aortic aneurysm; **EVAR:** endovascular aneurysm repair; **FEVAR:** fenestrated EVAR; **ChEVAR:** chimney EVAR; **OS:** open surgery; **JAAA/PAAA:** juxta/pararenal abdominal aortic aneurysm

Appendix SC (3). Summary of findings table showing comparison Chimney EVAR vs FEVAR for JAAA/PAAA treatment

Outcomes	**Anticipated absolute effects*** (95% CI)		Relative effect (95% CI)	№ of participants (studies)	Certainty of the evidence (GRADE)
	**Risk with FEVAR**	**Risk with ChEVAR**			
All-cause morality	123 per 1,000	**111 per 1,000** (65 to 178)	**OR 0.89** (0.50 to 1.55)	1679 (14 observational studies)	⊕⊕⊕◯ Moderate
Aortic-related reintervention	161 per 1,000	**122 per 1,000** (51 to 230)	**OR 0.72** (0.28 to 1.55)	749 (11 observational studies)	⊕⊕◯◯ Low
Aortic-related mortality	8 per 1,000	**13 per 1,000** (2 to 87)	**OR 1.55** (0.20 to 11.06)	701 (10 observational studies)	⊕◯◯◯ Very low
Branch/bypass occlusion/stenosis	41 per 1,000	**51 per 1,000** (14 to 178)	**OR 1.28** (0.34 to 5.11)	881 (13 observational studies)	⊕⊕◯◯ Low
Major adverse cardiovascular event	51 per 1,000	**192 per 1,000** (26 to 735)	**OR 4.39** (0.49 to 51.21)	399 (5 observational studies)	⊕◯◯◯ Very low
New onset renal replacement therapy	19 per 1,000	**15 per 1,000** (0 to 384)	**OR 0.82** (0.02 to 32.71)	296 (6 observational studies)	⊕◯◯◯ Very low
Total endoleak	148 per 1,000	**165 per 1,000** (71 to 378)	**OR 1.14** (0.44 to 3.51)	815 (12 observational studies)	⊕⊕◯◯ Low
Type I/III endoleak	57 per 1,000	**87 per 1,000** (30 to 246)	**OR 1.59** (0.52 to 5.43)	815 (11 observational studies)	⊕⊕◯◯ Low

AAA: abdominal aortic aneurysm; **EVAR:** endovascular aneurysm repair; **FEVAR:** fenestrated EVAR; **ChEVAR:** chimney EVAR; **OS:** open surgery; **JAAA/PAAA:** juxta/pararenal abdominal aortic aneury

Appendix SD. Model comparison of fixed-effect vs. random-effects models for the primary outcomes

	Mortality*		Aortic-related reintervention**		Aortic-related mortality***	
Summary	Fixed-effect model	Random-effects model	Fixed-effect model	Random-effects model	Fixed-effect model	Random-effects model
Posterior mean residual deviances, Dres	29.31	27.20	31.27	25.94	16.8	15.39
Effective number of parameters, pD	15.941	19.16	12.932	17.742	7.944	9.887
Between-study standard deviation, σ: posterior median (95% credible interval)	/	0.24 (0.01-0.71)	/	0.63 (0.04-1.81)	/	1.79 (0.08-4.77)
Deviance information criteria, DIC	154.815	155.918	113.971	113.448	45.740	46.275

*N=30 data points (12x2+2x3) **N=24 data points (9x2+2x3), ***N=15 data points (6x2+1x3)

Appendix SE. Sensitivity analysis including studies which included only patients with juxtarenal abdominal aortic aneurysms

	FEVAR vs. OS	ChEVAR vs. OS	ChEVAR vs. FEVAR	Heterogeneity	Number of studies
Mortality	**1.65 (1.08-2.33)**	1.43 (0.74-2.63)	0.87 (0.46-1.64)	0.21 (0.01-0.75)	12*
Aortic-related reintervention	**9.61 (3.44-46.22)**	**7.11 (2.06-32.67)**	0.75 (0.21-2.04)	0.87 (0.09-1.86)	9*
Aortic-related mortality	0.19 (0.01-2.53)	0.69 (0.04-7.57)	3.53 (0.36-46.76)	1.12 (0.07-1.96)	5*

Legend: values are presented as OR (95% CrI), the treatment stated first is the reference treatment, OR<1 favor the reference treatment, significant intervals are in bold; OS-open surgery, FEVAR-fenestrated endovascular repair, ChEVAR- chimney endovascular repair

*Studies Banno H et al. [21], and Fiorucci B et al. [27] have been excluded in the sensitivity analysis

The results stayed robust in the sensitivity analysis.

Appendix SF. The probability of each treatment being the best, second best, and worst for primary outcomes

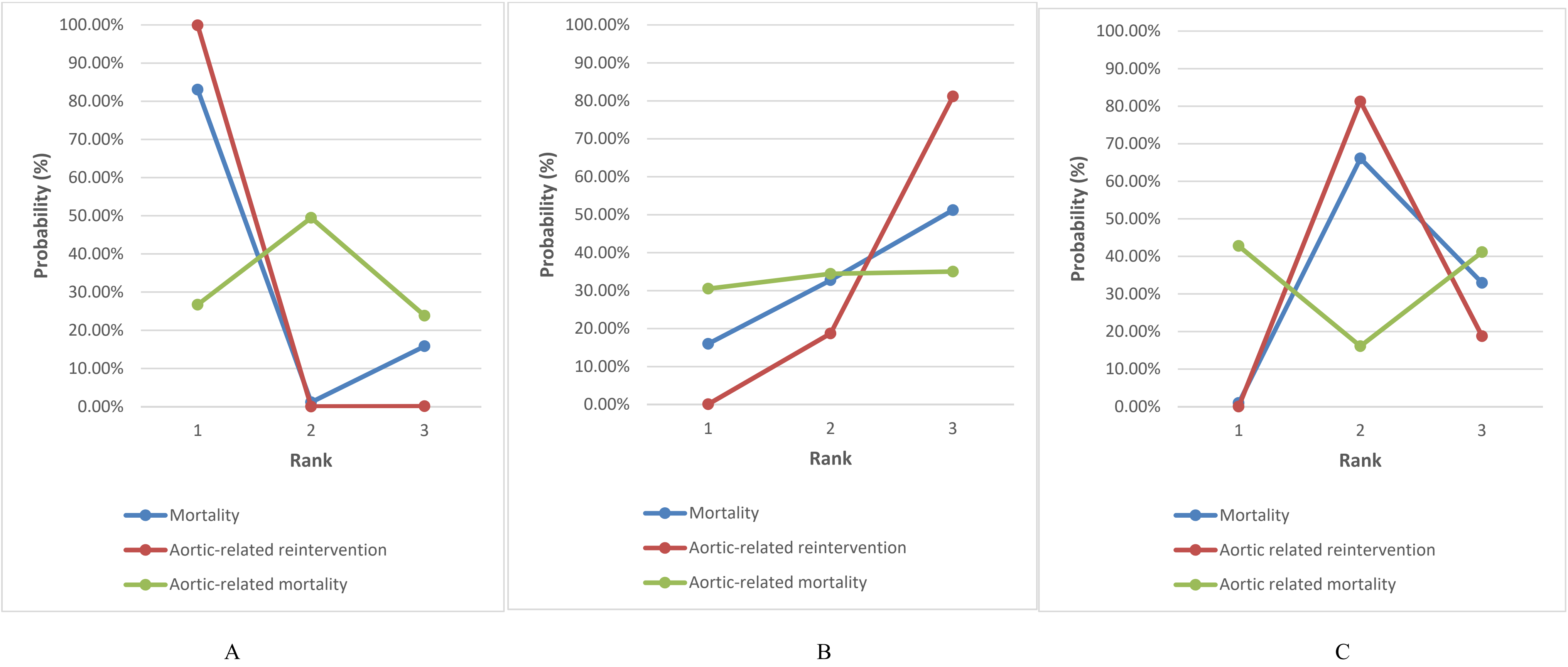

Legend. Probability of open surgery being ranked #1, #2, or #3 compared to FEVAR and ChEVAR (A); Probability of FEVAR being ranked #1, #2, or #3 compared to open surgery and ChEVAR (B); Probability of ChEVAR being ranked #1, #2, or #3 compared to open surgery and FEVAR (C); OS-open surgery, FEVAR-fenestrated endovascular repair, ChEVAR- chimney endovascular repair

Appendix SG. Definitions of JAA/PAAA patients in included studies.

First Author (Year of publication)	AAA type	Intervention	JAAA/PAAA definition
Donas et al. (2012) [17]	Juxtarenal	ChEVAR<br>FEVAR<br>Open surgery	Primary degenerative JAAA - a complex abdominal aortic aneurysm with a short infrarenal neck (<9 mm) or aneurysmal extension to the inter-renal aorta.
Wei et al. (2013) [18]	Juxtarenal	ChEVAR<br>FEVAR	No definition provided.
Lee et al. (2014) [19]	Juxtarenal	ChEVAR<br>FEVAR	No definition provided.
Barilla et al. (2014) [20]	Juxtarenal	Open surgery<br>FEVAR	Length shorter than 10 mm, diameter larger than 31 mm not suitable for standard EVAR. Exclusion criteria were the involvement of the celiac trunk (type IV TAAA were thus excluded), and an emergency setting (tender and ruptured AAA).
Banno et al. (2014) [21]	Juxta/ pararenal	FEVAR<br>ChEVAR	Juxtarenal and PAAs considered unsuitable for conventional EVAR. Exclusion criteria were bailout chimney graft procedures for unexpected covering of one or both renal arteries during standard EVAR, f-EVAR with a physician-modified stent graft, and branched or combined (ie, fenestrated and branched or fenestrated and chimney) stent grafts. Patients with failure of cannulation through fenestration(s), followed by reconstruction of visceral branch(es), by bailout chimney technique were included in the f-EVAR cohort.
Shahverdyan et al. (2015) [22]	Juxtarenal	Open surgery<br>FEVAR	The definition of JAA was based on the short necked AAA, if the CC was needed above one or both renal arteries in the OSR group. The choice for F-EVAR was based on a short infrarenal aneurysm neck of ≤10 mm.
Saratzis et al. (2015) [23]	Juxtarenal	FEVAR<br>Open surgery	No definition provided.
Maeda et al. (2015) [24]	Juxtarenal	Open surgery<br>FEVAR<br>ChEVAR	JAAA was defined as juxtarenal aneurysms with a short proximal neck less than 1.0 cm. Aneurysm involving the superior mesenteric artery (SMA) or the celiac artery were classified as Crawford type 4 thoracoabdominal aortic aneurysm and were excluded from this study.
Wooster et al. (2016) [25]	Juxtarenal	ChEVAR<br>FEVAR	Juxtarenal aneurysm - aneurysmal dilatation extending to within 4 mm of the lowest renal artery but not more proximal than the highest renal artery. Those treated for rupture were excluded.

Caradu et al. (2017) [26]	Juxtarenal	FEVAR<br>ChEVAR	No definition provided.
Fiorucci et al. (2018) [27]	Pararenal	FEVAR<br>Open surgery	No definition provided.
Chinsakchai et al. (2019) [28]	Juxtarenal	Open surgery<br>FEVAR<br>ChEVAR	Inclusion criteria were AAA with a short neck for endovascular treatment not suitable for standard EVAR, and no normal aorta available for proximal clamping between the upper extent of the aneurysm and the renal artery for OR. The indications for treatment included the large fusiform JAAA (> 5 cm) or a saccular aneurysm with a short proximal sealing zone for standard EVAR. Patients with ruptured JAAA were excluded
Soler et al. (2019) [29]	Juxtarenal	Open surgery<br>FEVAR	Type A corresponds to an aneurysm of the inter-renal aorta, more or less extending to the suprarenal aorta; this dilation develops at the posterior aortic wall. Type B describes a normal inter-renal aorta but aneurysmal involvement of one or two renal artery origins. Type C is a JRA with a normal inter-renal aorta and absence of renal artery aneurismal disease.
O'Donnell et al. (2020) [30]	Juxtarenal	Open surgery<br>FEVAR	For FEVAR, we defined juxtarenal AAA as having a proximal extent at or below the highest renal artery where at least 1 renal artery was incorporated into the repair with a branch or fenestration. In the open repair cohort, we included all AAA in which the surgeon recorded placing a clamp above at least 1 renal artery. Any procedure that involved a chimney or snorkel was excluded. The VQI does not report outcomes from patients enrolled in IDE studies or pivotal trials, so only commercially available FEVAR devices are included. We excluded patients who underwent FEVAR after a visceral debranching procedure. For open repairs, we excluded patients with prior abdominal or thoracoabdominal aortic surgery or prior EVAR to avoid including graft explants and reoperations for complications
Menegolo et al. (2021) [31]	Juxtarenal	ChEVAR<br>Open surgery	JAAA was defined as an aneurysm extending up to but not involving the renal arteries, necessitating transrenal or suprarenal aortic clamping for OSR (e.g. a short neck < 10 mm or sealing zone $\leqslant$4 mm)
Bootun et al. (2021) [32]	Juxtarenal	Open surgery<br>FEVAR	No definition provided.

Legend. AAA- abdominal aortic aneurysm, JAAA – juxtarenal abdominal aortic aneurysm, PAAA – pararenal abdominal aortic aneurysm, FEVAR-fenestrated endovascular repair, ChEVAR- chimney endovascular repair
